# Supplementary material for: Human Erbb2-induced Erk activity robustly stimulates cycling and functional remodeling of rat and human cardiomyocytes
Source: eLife. 2021 Oct 19;10:e65512. doi: 10.7554/eLife.65512 (PMC8589446; doi:10.7554/eLife.65512)
Supplement: Supplementary file 1. [file elife-65512-supp1.docx]

**Supplementary File 1. Statistical Information for all Figures**

| Figure | n | Definition of n | Statistical test |
| --- | --- | --- | --- |
| 1B | 9-28 | One CM monolayer, 10,000 cells by FACS | Welch’s ANOVA on the logarithmically-transformed FC data values followed by post-hoc tests with Dunnett T3 corrections for multiple comparisons, vs. Ctrl LV |
| 1C | 5-21 | One CM monolayer, 10,000 cells by FACS | Welch’s ANOVA on the raw data values followed by post-hoc tests with Dunnett T3 corrections for multiple comparisons, vs. Ctrl LV |
| 1D-E | 12-30 | One CM monolayer, 10,000 cells by FACS | One-way ANOVA on the raw data values followed by Dunnett post-hoc tests vs. Ctrl LV |
| 1F | 6 | One CM monolayer, 10,000 cells by FACS | One-way ANOVA on the raw data values followed by Holm-Sidak post-hoc tests vs. Ctrl LV |
| 1G | 8-9 | RNA isolated from one CM monolayer | One-way ANOVA on the logarithmically-transformed FC data values followed by Holm-Sidak post-hoc tests vs. Ctrl LV |
| 1H | 6-8 | Protein isolated from one CM monolayer | One-way ANOVA on the logarithmically-transformed FC data values followed by Holm-Sidak post-hoc tests vs. Ctrl LV |
| 1J | 3 | One CM monolayer | Unpaired t test comparing logarithmically-transformed FC data values |
| 1K | 4-7 | RNA isolated from one CM monolayer | One-way ANOVA on the logarithmically-transformed FC data values followed by Holm-Sidak post-hoc tests vs. Ctrl LV |
| 1L | 9 | Protein isolated from one CM monolayer | Unpaired t test comparing logarithmically-transformed FC data values |
| 1M | 8-9 | RNA isolated from one CM monolayer | Unpaired t test comparing logarithmically-transformed FC data values |
| 1N | 7-9 | Protein isolated from one CM monolayer | Unpaired t test comparing logarithmically-transformed FC data values |
| 1P | 3 | One CM monolayer | Unpaired t test comparing logarithmically-transformed FC data values |
| 2B-C | 6-18 | One CM monolayer, 10,000 cells by FACS | Welch’s ANOVA on the logarithmically-transformed FC data values followed by post-hoc tests with Dunnett T3 corrections for multiple comparisons, vs. Ctrl LV |
| 2D-E | 6-16 | One CM monolayer, 10,000 cells by FACS | One-way ANOVA on the logarithmically-transformed data values followed by Holm-Sidak post-hoc tests vs. Ctrl LV. |
| 2G | 7-9 | RNA isolated from one CM monolayer | One-way ANOVA on the logarithmically-transformed FC data values followed by Holm-Sidak post-hoc tests vs. Ctrl LV |
| 3C | 8-40 | One cardiobundle | Welch’s ANOVA on the raw data values followed by post-hoc tests with Dunnett T3 corrections for multiple comparisons, vs. Ctrl LV. |
| 3D-F | 8-44 | One cardiobundle | Welch’s ANOVA on the logarithmically-transformed data values followed by post-hoc tests with Dunnett T3 corrections for multiple comparisons, vs. Ctrl LV. |
| 3G | 8-15 | One cardiobundle | One-way ANOVA on the raw data values followed by Holm-Sidak post-hoc tests vs. Ctrl LV |
| 3H | 12-34 | One cardiobundle | Welch’s ANOVA on the raw data values followed by post-hoc tests with Dunnett T3 corrections for multiple comparisons, vs. Ctrl LV. |
| 3I | 11-34 | One cardiobundle | Line plot: Multiple unpaired t-tests, Holm-Sidak correction for multiple comparisons using a statistical significance approach to determine p values. Bar graph: Welch’s ANOVA on the raw data values followed by post-hoc tests with Dunnett T3 corrections for multiple comparisons, vs. Ctrl LV |
| 4A | 12-21 | Protein isolated from one CM monolayer | Two-way ANOVA on the logarithmically-transformed FC data values followed by Holm-Sidak post-hoc tests vs. Ctrl LV |
| 4B | 6-9 | RNA isolated from one CM monolayer | One-way ANOVA on the logarithmically-transformed FC data values followed by Holm-Sidak post-hoc tests vs. Ctrl LV |
| 4C | 5 | One CM monolayer | Welch’s t test comparing logarithmically-transformed FC values. |
| 4D | 8-9 | RNA isolated from one CM monolayer | One-way ANOVA on the logarithmically-transformed FC data values followed by Holm-Sidak post-hoc tests vs. Ctrl LV |
| 4E | 13 | One CM monolayer | Welch’s t test comparing logarithmically-transformed FC values. |
| 4F | 6-9 | Protein isolated from one CM monolayer | Two-way ANOVA on the logarithmically-transformed FC data values followed by Holm-Sidak post-hoc tests vs. Ctrl LV |
| 4G | 7 | One CM monolayer | Welch’s t test comparing raw data values. |
| 5C | 8-16 | One cardiobundle | Welch’s ANOVA on the raw data values followed by post-hoc tests with Dunnett T3 corrections for multiple comparisons, vs. Ctrl LV. |
| 5D-F | 8-22 | One cardiobundle | Welch’s ANOVA on the raw data values followed by post-hoc tests with Dunnett T3 corrections for multiple comparisons, comparing all groups. |
| 5G | 18-35 | One cardiobundle | Welch’s ANOVA on the raw data values followed by post-hoc tests with Dunnett T3 corrections for multiple comparisons, comparing all groups |
| 5H | 18-32 | One cardiobundle | Line plot: Multiple unpaired t-tests, Holm-Sidak correction for multiple comparisons using a statistical significance approach to determine p values. Bar graph: Welch’s ANOVA on the raw data values followed by Holm-Sidak post-hoc tests vs. Ctrl LV |
| 1-S1B | 15-38 | One CM monolayer, 10,000 cells by FACS | One-way ANOVA on the raw data values followed by Holm-Sidak post-hoc tests vs. Ctrl LV |
| 1-S1C | 6-18 | One CM monolayer, 10,000 cells by FACS | One-way ANOVA on the raw data values followed by Holm-Sidak post-hoc tests vs. Ctrl LV |
| 1-S2B  (EdU) | 12-28 | One CM monolayer, 10,000 cells by FACS | One-way ANOVA on the logarithmically-transformed FC data values followed by Holm-Sidak post-hoc tests vs. Ctrl LV |
| 1-S2B  (H3P) | 14 | One CM monolayer, 10,000 cells by FACS | Unpaired t test comparing raw values |
| 1-S2B  (Cc3) | 6 | One CM monolayer, 10,000 cells by FACS | Unpaired t test comparing raw values |
| 1-S3B | 12 | One CM monolayer, 10,000 cells by FACS | Welch’s ANOVA on the logarithmically-transformed FC data values followed by post-hoc tests with Dunnett T3 corrections for multiple comparisons, vs. Ctrl LV |
| 1-S3C | 12 | One CM monolayer, 10,000 cells by FACS | Unpaired t test comparing logarithmically-transformed FC values. |
| 1-S3D | 12 | One CM monolayer, 10,000 cells by FACS | Unpaired t test comparing raw values |
| 2-S1A | 4-8 | One CM monolayer, 10,000 cells by FACS | Unpaired t test comparing raw values |
| 2-S1B  (left) | 21-23 | One CM monolayer, 10,000 cells by FACS | Welch’s t test on the logarithmically-transformed FC data values |
| 2-S1B  (right) | 6 | One CM monolayer, 10,000 cells by FACS | Welch’s t test on the logarithmically-transformed FC data values |
| 3-S1B-C | 20-44 | One cardiobundle | Unpaired t test comparing raw values |
| 3-S1D | 34 | One cardiobundle | Unpaired t test comparing raw values |
| 3-S1E | 25-30 | One cardiobundle | Line plot: Multiple unpaired t-tests, Holm-Sidak correction for multiple comparisons using a statistical significance approach to determine p values. Bar graph: unpaired t test comparing raw data values |
| 3-S2A-C | 16-37 | One cardiobundle | Welch’s ANOVA on the raw data values followed by post-hoc tests with Dunnett T3 corrections for multiple comparisons, vs. Ctrl LV |
| 3-S2D | 8-15 | One cardiobundle | One-way ANOVA on the raw data values followed by Holm-Sidak post-hoc tests vs. Ctrl LV |
| 3-S2E | 6 | One cardiobundle | Unpaired t test comparing raw values |
| 3-S3A | 10-12 | One cardiobundle | Welch’s t test comparing logarithmically-transformed FC data values |
| 3-S3B | 9 | One cardiobundle | Unpaired t test comparing raw values |
| 3-S3C | 8-11 | One cardiobundle | Welch’s t test comparing raw values |
| 3-S3D | 32-44 | One cardiobundle | Welch’s t test comparing raw values |
| 3-S3E | 5-6 | One cardiobundle | Unpaired t test comparing raw values |
| 5-S1B-C | 9 | One CM monolayer, 10,000 cells by FACS | One-way ANOVA on the logarithmically-transformed FC data values followed by Holm-Sidak post-hoc tests comparing all groups |
| 5-S2A-C | 18-32 | One cardiobundle | Welch’s ANOVA on the raw data values followed by post-hoc tests with Dunnett T3 corrections for multiple comparisons. comparing all groups. |
