# Supplementary material for: Human Erbb2-induced Erk activity robustly stimulates cycling and functional remodeling of rat and human cardiomyocytes
Source: eLife. 2021 Oct 19;10:e65512. doi: 10.7554/eLife.65512 (PMC8589446; doi:10.7554/eLife.65512)
Supplement: Source data 2. [file elife-65512-supp3.docx]

Raw unedited labeled Western blot images

| Figure 1H |  | Control | | |  | Yap8SA | | |  | | |
| --- | --- | --- | --- | --- | --- | --- | --- | --- | --- | --- | --- |
|  |  | 1 | 2 | 3 |  | 4 | 5 | 6 |  | |  |
| Active Yap  GAPDH | 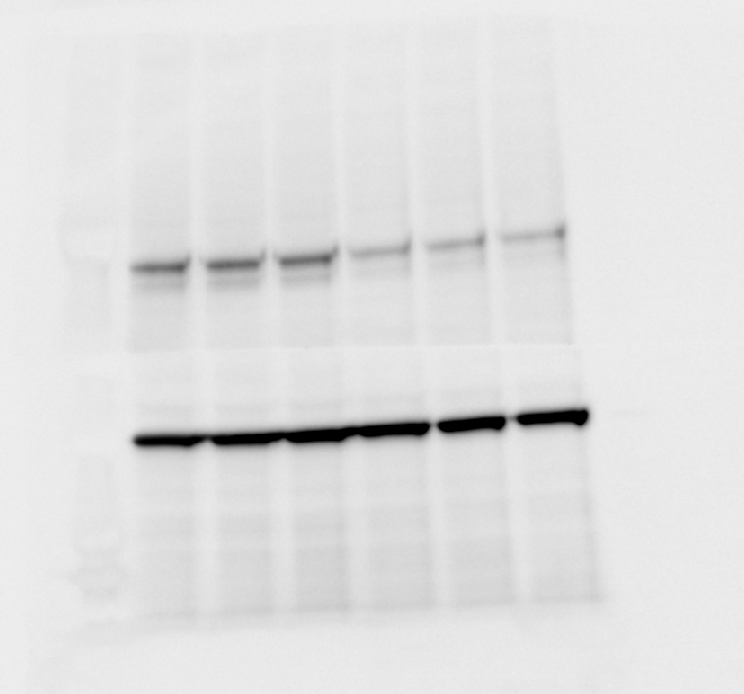 | | | | | | | | |  |  |

| Figure 1H |  | Control LV | | | caYap8SA | | |  | |
| --- | --- | --- | --- | --- | --- | --- | --- | --- | --- |
|  |  | 1 | 2 | 3 | 4 | 5 | 6 |  | |
| Total Yap  GAPDH | 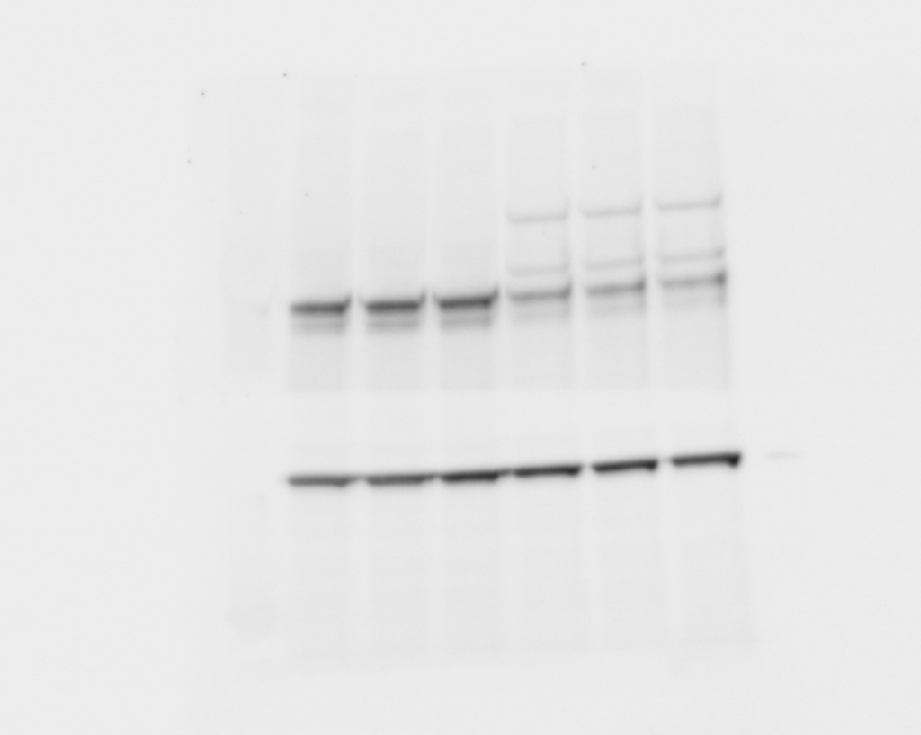 | | | | | | | |  |

| Figure 1L |  | | cahErbb2 | | | Control LV | | | caCtnnb1 | | |  | | |
| --- | --- | --- | --- | --- | --- | --- | --- | --- | --- | --- | --- | --- | --- | --- |
|  |  | 1 | | 2 | 3 | 4 | 5 | 6 | 7 | 8 | 9 |  | |  |
| ß-catenin  GAPDH | 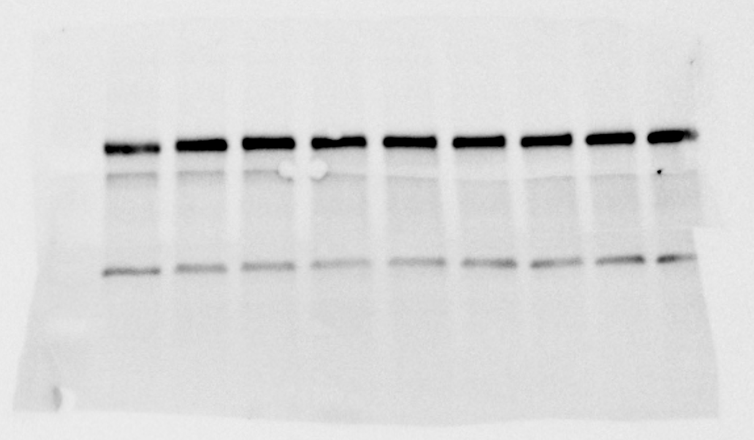 | | | | | | | | | | | |  |  |

| Figure 1N | cahErbb2 | | | | Control LV | | | Ccnd2 OE | | | |  | | |
| --- | --- | --- | --- | --- | --- | --- | --- | --- | --- | --- | --- | --- | --- | --- |
|  |  | 1 | 2 | 3 | 4 | 5 | 6 | 7 | 8 | 9 |  | | |  |
| CyclinD2 | 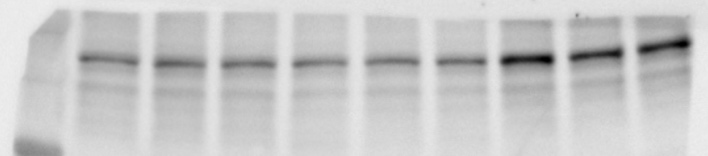 | | | | | | | | | | | |  |  |
| GAPDH | 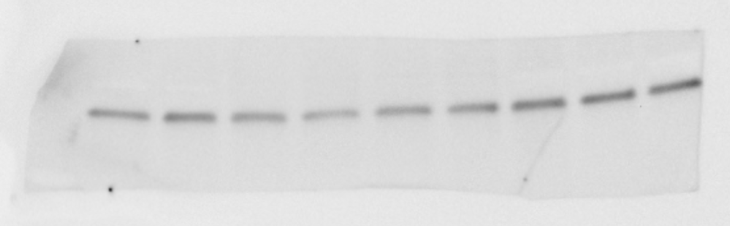 | | | | | | | | | | | |  |  |

| Figure 4A | Control LV | | | | carErbb2 | | | cahErbb2 | | |  | |
| --- | --- | --- | --- | --- | --- | --- | --- | --- | --- | --- | --- | --- |
|  |  | 1 | 2 | 3 | 4 | 5 | 6 | 7 | 8 | 9 |  |  |
| pAkt  Erk | 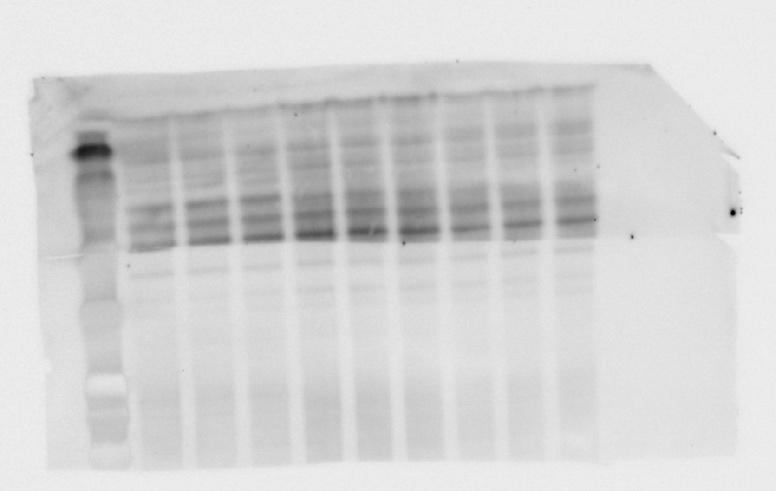 | | | | | | | | | | |  |
| Akt  GAPDH | 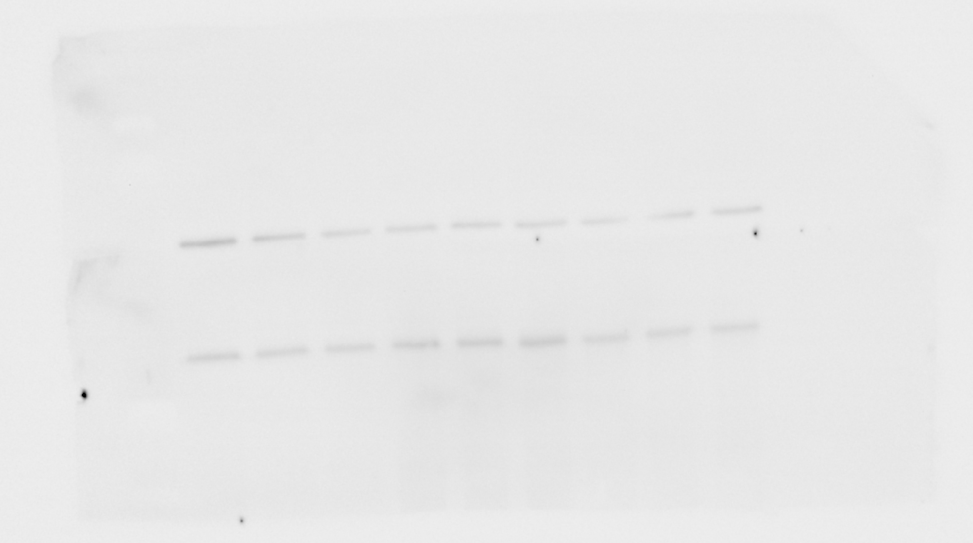 | | | | | | | | | | |  |
| pErk | 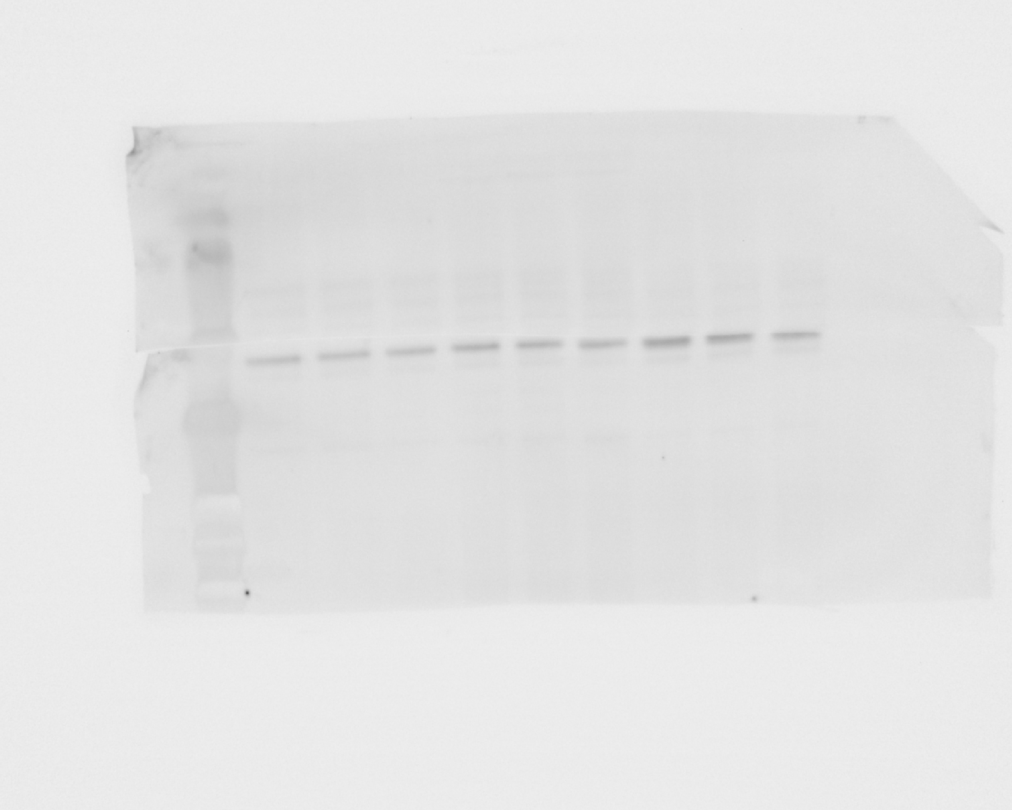 | | | | | | | | | | |  |

| Figure 4F | Control LV | | | | carErbb2 | | | cahErbb2 | | |  |
| --- | --- | --- | --- | --- | --- | --- | --- | --- | --- | --- | --- |
|  |  | 1 | 2 | 3 | 4 | 5 | 6 | 7 | 8 | 9 |  |
| pmTOR  pS6 | 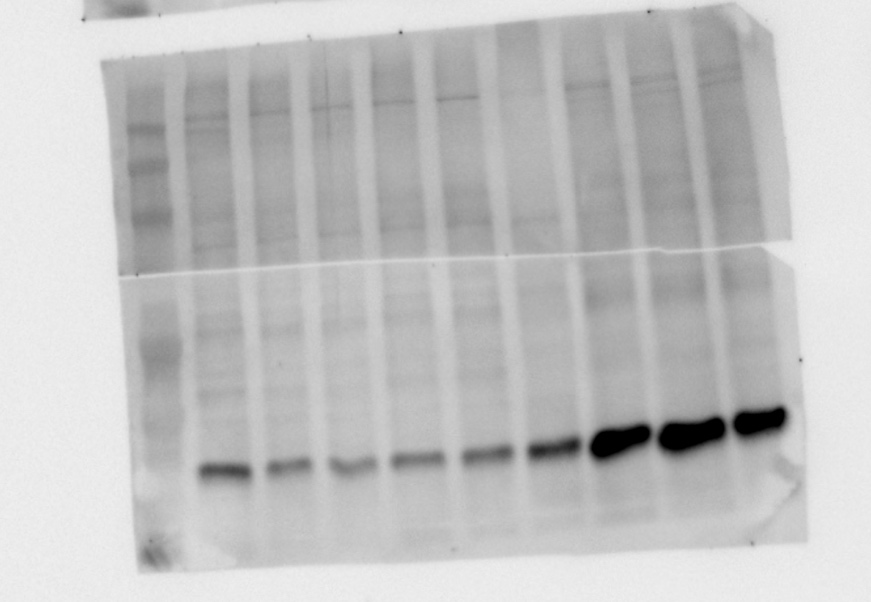 | | | | | | | | | | |
| GAPDH | 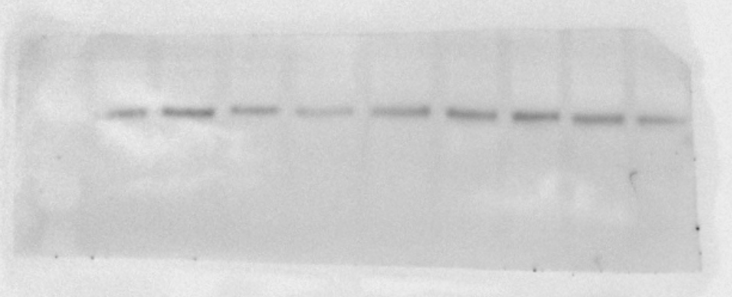 | | | | | | | | | | |
