# Supplementary figures and images for: Human Erbb2-induced Erk activity robustly stimulates cycling and functional remodeling of rat and human cardiomyocytes
Source: eLife. 2021 Oct 19;10:e65512. doi: 10.7554/eLife.65512 (PMC8589446; doi:10.7554/eLife.65512)

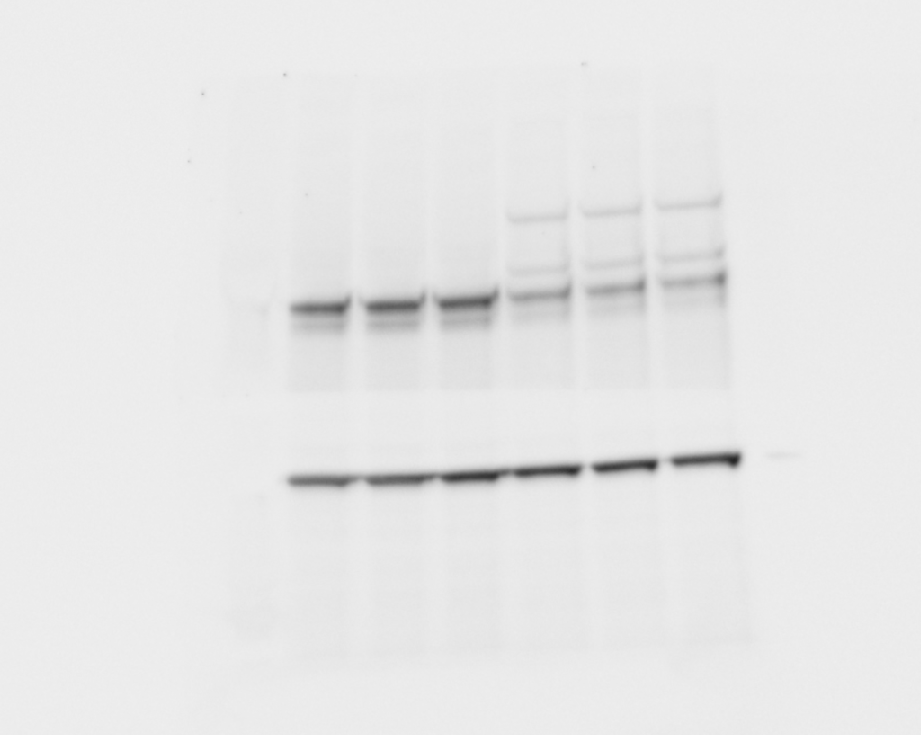

Supplement: Source data 3. [file elife-65512-supp4.zip › Raw blots images for Elife/Fig 1H Total Yap.tif]

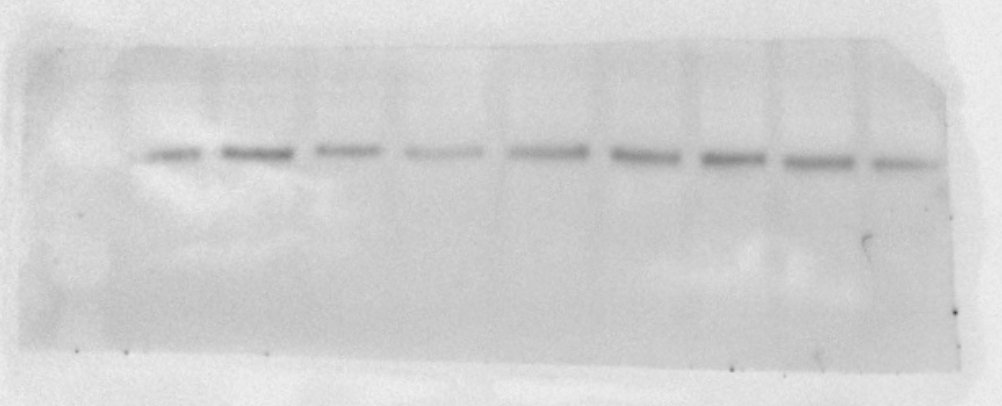

Supplement: Source data 3. [file elife-65512-supp4.zip › Raw blots images for Elife/Fig 4F GAPDH.tif]

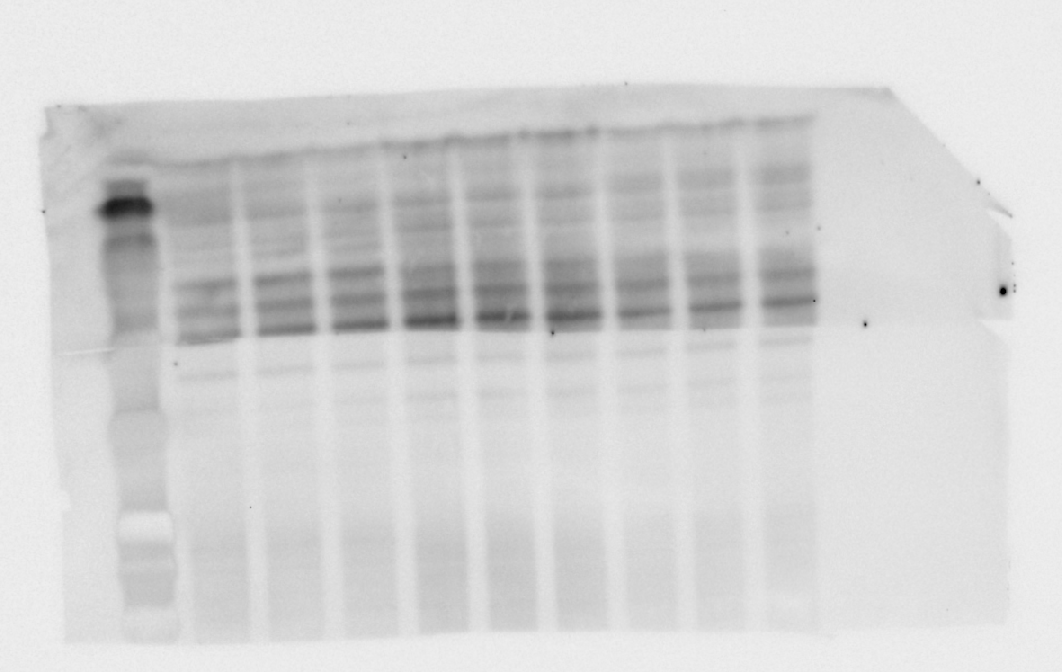

Supplement: Source data 3. [file elife-65512-supp4.zip › Raw blots images for Elife/Fig 4A pAkt and Erk.tif]

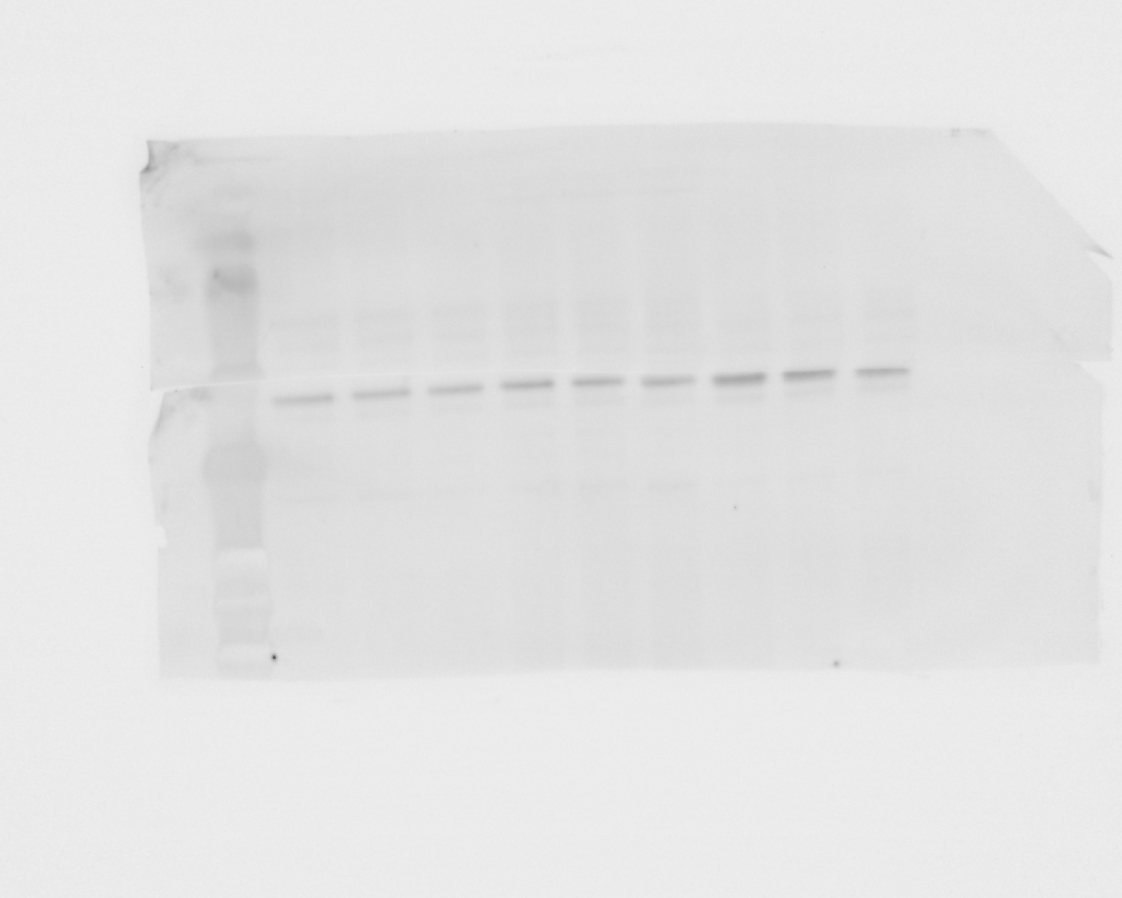

Supplement: Source data 3. [file elife-65512-supp4.zip › Raw blots images for Elife/Fig 4A pErk.tif]

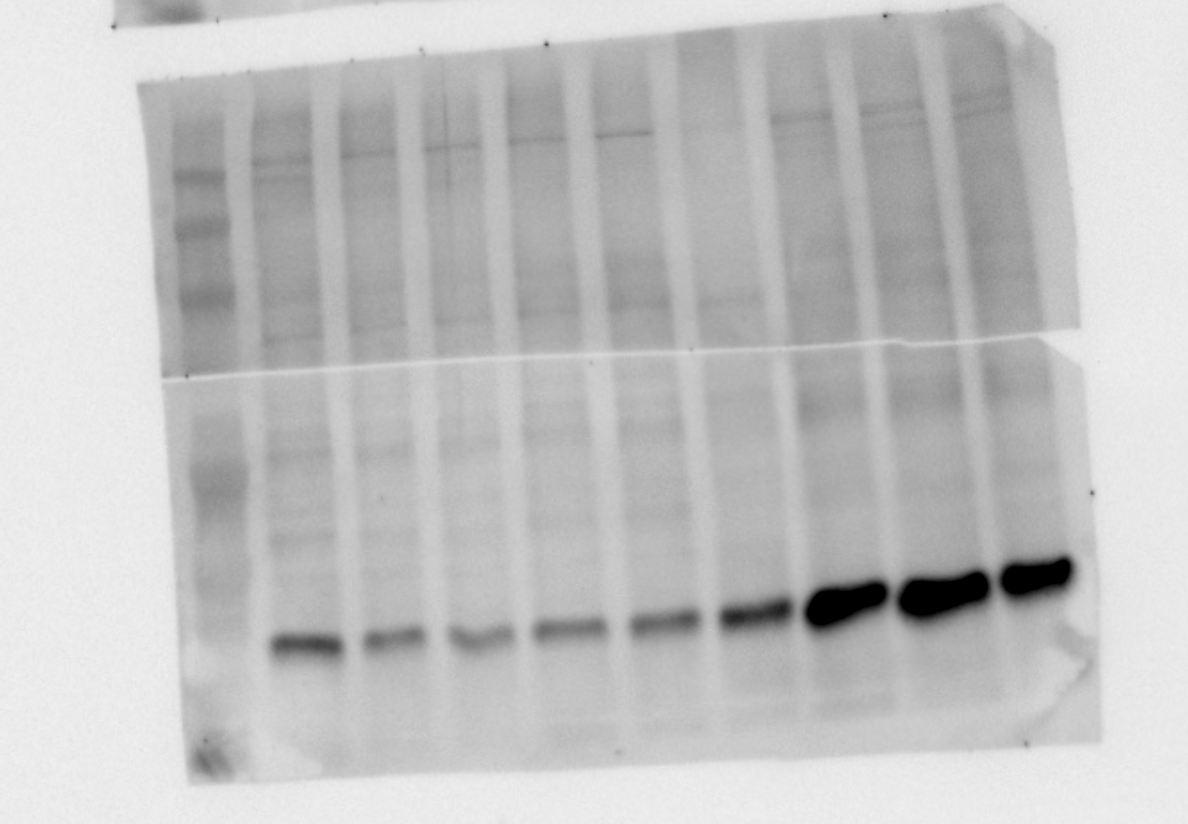

Supplement: Source data 3. [file elife-65512-supp4.zip › Raw blots images for Elife/Fig 4F pmTOR and pS6.tif]

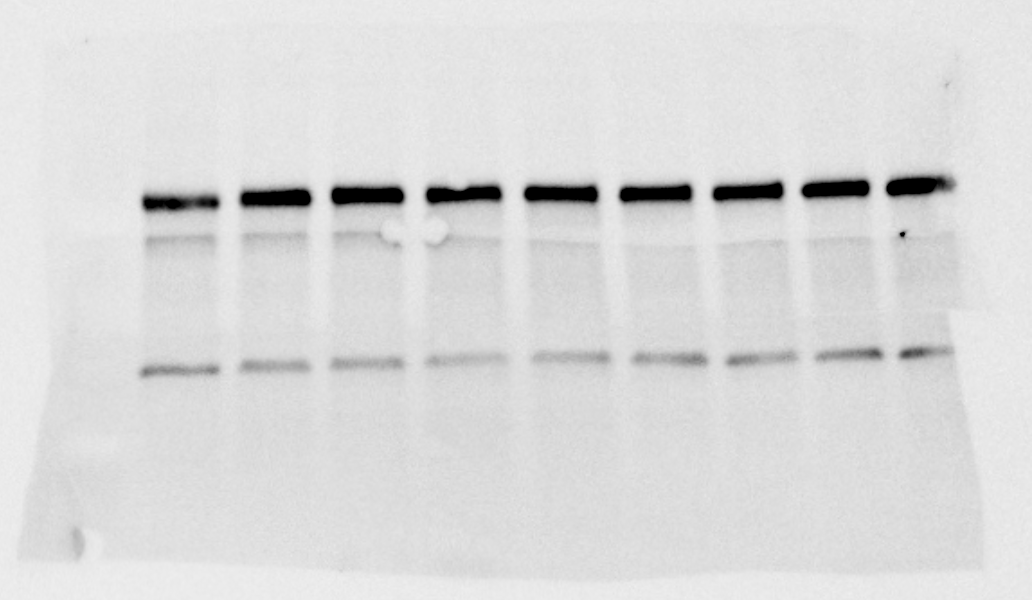

Supplement: Source data 3. [file elife-65512-supp4.zip › Raw blots images for Elife/Fig 1L Bcatenin and GAPDH.tif]

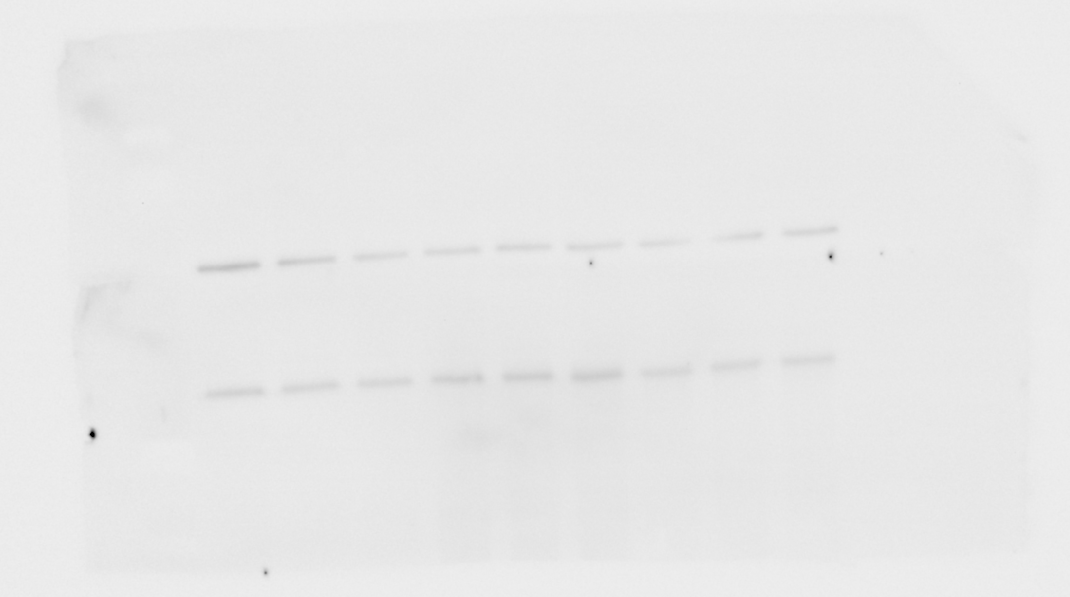

Supplement: Source data 3. [file elife-65512-supp4.zip › Raw blots images for Elife/Fig 4A Akt and GAPDH.tif]

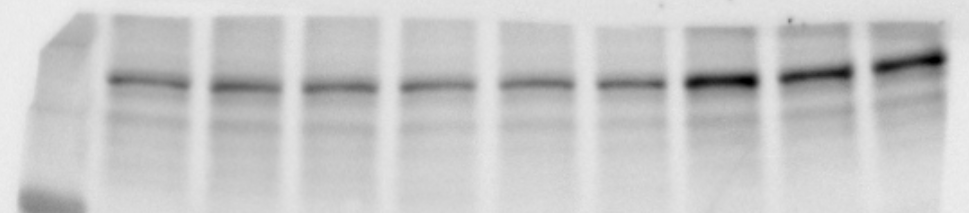

Supplement: Source data 3. [file elife-65512-supp4.zip › Raw blots images for Elife/Fig 1N CyclinD2.tif]

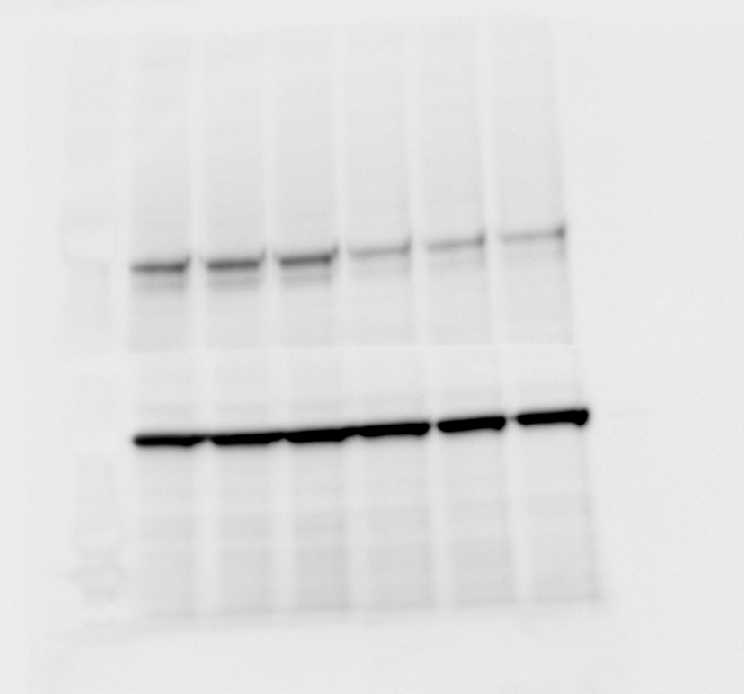

Supplement: Source data 3. [file elife-65512-supp4.zip › Raw blots images for Elife/Fig 1H Active Yap and GAPDH.tif]

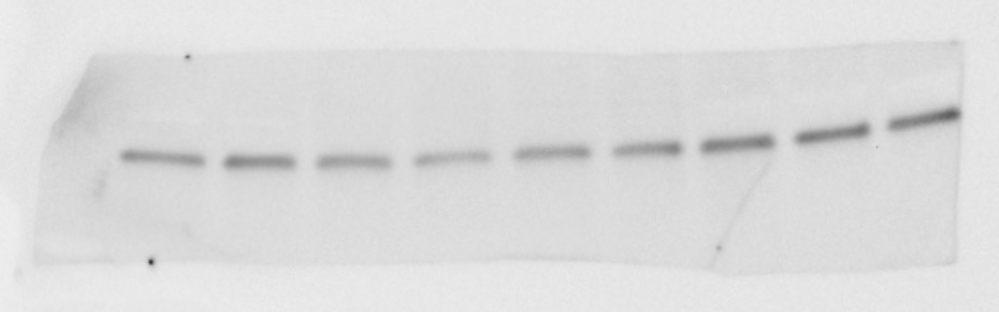

Supplement: Source data 3. [file elife-65512-supp4.zip › Raw blots images for Elife/Fig 1N GAPDH.tif]
